# THREE MONTHS OF STRENGTH TRAINING ALTERS LPS-INDUCED IMMUNE RESPONSES IN CIRCULATING PBMC OF OLDER PERSONS

**DOI:** 10.1093/geroni/igad104.0133

**Published:** 2023-12-21

## Abstract

BACKGROUND Recently, we showed that 3 months strength training affect 18 canonical pathways related to chronic inflammation in peripheral mononuclear blood cells (PBMC) of older adults. Here we investigate if resistance training improves the stress response of PBMC by mimicking in-vitro an acute infection by lipopolysaccharide (LPS)-challenge. METHODS 14 women aged ≥65 years were randomized into 3 months of either 3×/week intensive strength training (IST: 3×10 rep at 80% 1RM), strength endurance training (SET: 2×30 reps at 40% 1RM) or control (CON: 3×30 sec stretching). Before and after 3 months training, PBMC were isolated and cultured with and without LPS. Prior culture and after 24 hours of culture, RNA was collected from pre-cultured, post-cultured and LPS challenged PBMC’s, respectively. Targeted RNA sequencing including 407 inflammation-related genes was performed. Pathway analysis was performed with Ingenuity Pathway Analyses using all 407 genes, a Benjamini-Hochberg p-value < 0.05 and a z-score of ≤-2 or ≥2 were considered as significant. RESULTS Strength training altered 23 pathways in LPS-stimulated PBMC; (IST: 7 upregulated and 2 down-regulated, SET: 5 upregulated and 10 downregulated). None of the altered pathways overlapped between IST and SET. The Cytotoxic T Lymphocyte-mediated Apoptosis of Target Cells pathway was enriched oppositely in both training groups (downregulated in IST versus upregulated in SET). CONCLUSIONS Three months IST and SET can induce changes in the inflammatory stress response of PBMC, but by affecting different genes and related pathways. A balanced exercise program altering both training regimes might therefore provide optimal immune adaptations in older persons.

